# Creation and validation of models to predict response to primary treatment in serous ovarian cancer

**DOI:** 10.1038/s41598-021-85256-9

**Published:** 2021-03-16

**Authors:** Jesus Gonzalez Bosquet, Eric J. Devor, Andreea M. Newtson, Brian J. Smith, David P. Bender, Michael J. Goodheart, Megan E. McDonald, Terry A. Braun, Kristina W. Thiel, Kimberly K. Leslie

**Affiliations:** 1 Division of Gynecologic Oncology, Department of Obstetrics and Gynecology, University of Iowa Hospitals and Clinics, Iowa City, IA 52242 USA; 2 Holden Comprehensive Cancer Center, University of Iowa Hospitals and Clinics, Iowa City, IA 52242 USA; 3 Department of Obstetrics and Gynecology, University of Iowa Hospitals and Clinics, Iowa City, IA 52242 USA; 4 Department of Biostatistics, University of Iowa College of Public Health, Iowa City, IA 52242 USA; 5 Coordinated Laboratory for Computational Genomics, University of Iowa Hospitals and Clinics, Iowa City, IA 52242 USA

**Keywords:** Cancer, Cancer genomics

## Abstract

Nearly a third of patients with high-grade serous ovarian cancer (HGSC) do not respond to initial therapy and have an overall poor prognosis. However, there are no validated tools that accurately predict which patients will not respond. Our objective is to create and validate accurate models of prediction for treatment response in HGSC. This is a retrospective case–control study that integrates comprehensive clinical and genomic data from 88 patients with HGSC from a single institution. Responders were those patients with a progression-free survival of at least 6 months after treatment. Only patients with complete clinical information and frozen specimen at surgery were included. Gene, miRNA, exon, and long non-coding RNA (lncRNA) expression, gene copy number, genomic variation, and fusion-gene determination were extracted from RNA-sequencing data. DNA methylation analysis was performed. Initial selection of informative variables was performed with univariate ANOVA with cross-validation. Significant variables (p < 0.05) were included in multivariate lasso regression prediction models. Initial models included only one variable. Variables were then combined to create complex models. Model performance was measured with area under the curve (AUC). Validation of all models was performed using TCGA HGSC database. By integrating clinical and genomic variables, we achieved prediction performances of over 95% in AUC. Most performances in the validation set did not differ from the training set. Models with DNA methylation or lncRNA underperformed in the validation set. Integrating comprehensive clinical and genomic data from patients with HGSC results in accurate and robust prediction models of treatment response.

## Introduction

Despite notable advances in the treatment of ovarian cancer, it continues to be one of the leading causes of cancer death among women in the United States^1^. The most common type of ovarian cancer is high-grade serous cancer (HGSC). HGSC typically presents as advanced disease, and standard treatment consists of combined primary cytoreductive surgery and platinum-based chemotherapy^2^. Platinum is considered the most effective drug for HGSC^2^. Patients that respond to initial therapy and have progression-free survival (PFS) of at least 6 months are termed “platinum-sensitive” or “responders” and have a median survival of well over four years^3^. In patients that have no residual disease after the initial surgery and respond to chemotherapy, median survivals can reach over 10 years^3^. However, in nearly a third of patients, HGSC progresses during initial chemotherapy (termed “platinum-refractory”) or recurs < 6 months after finishing treatment (termed “platinum-resistant”)^2,4–6^. The majority of these patients with suboptimal response to initial treatment (termed “non-responders” herein) will die from their disease within two years^4,7,8^ and are typically treated in the second-line setting with alternative therapies that do not contain platinum^2^.

In recent years, significant efforts have been dedicated to test new targeted drugs in clinical trials to increase PFS of the patients that already respond to primary chemotherapy, with celebrated successes^9–12^. However, few resources have been dedicated to identify those patients that are at risk of failing initial treatment before its administration, and there is no validated test that can predict robustly and accurately this outcome^13,14^. By contrast, in breast cancer gene signatures have been identified that can accurately predict recurrence^15^ and chemotherapeutic response^16,17^. These signatures have been validated in independent clinical studies^17–20^. For example, one of these signatures, OncotypeDx, used 600 cases to create an association model and validated the model in an additional 400 cases^15,16^. The majority of previous attempts to define predictors of treatment response in HGSC have been limited by a small number of patients, mixture of histological types and stages, and lack of validation in independent datasets^13,14^. One of the more successful efforts used serum markers, including kallikreins and CA 125^13,14^. The performance of these prediction models ranges from 75–85% (measured as the area under the receiver operator curve (AUC)). Adding clinical characteristics to serum markers increases the performance of the model to an AUC of 90%^14^. Others have integrated the Cancer Genome Atlas (TCGA) genomic data to predict overall survival (OS) and PFS, with performances that ranged from AUCs of 81 to 87%^21^. However, none of these models have been validated in independent datasets, nor have they been validated prospectively.

Using publicly available multi-dimensional datasets with clinical data, like TCGA, we previously built prediction models that distinguish between different outcomes in ovarian and endometrial cancer; we validated these models in independent datasets^22–27^. However, these models had some limitations due to suboptimal clinical data: many patients were lost to follow-up, and others had little information about clinical variables that influenced treatment response, such as stage of disease or number of cycles of chemotherapy. Also, some datasets did not have complete molecular information because expression analyses were performed on different platforms, with different probes. This last limitation severely impacted the performance of the validation studies^22^.

Herein we tested the hypothesis that integrating comprehensive clinical and genomic data from patients with HGSC will ultimately result in a more accurate and robust prediction models of response to treatment. The primary objective of our study was to create models of prediction to standard therapy in patients with HGSC. The secondary objective was to validate these models in an independent dataset. Also, we intend to extract maximum genomic information from RNA-sequencing (RNA-seq) so resulting models would be feasible and affordable for any laboratory.

## Methods

This is a retrospective case–control study that used clinical and genomic information to create models to predict initial response to standard therapy for HGSC patients. The prediction was made using only data that could be obtained before the administration of the initial chemotherapy. Also, as we mentioned, we intend to extract maximum genomic information from RNA-sequencing (RNA-seq) experiments.

### Outcomes definition

HGSC patients were classified as responders or non-responders. Responders were those with a progression-free survival of at least 6 months after the first platinum-based treatment. Non-responders were those who did not respond (platinum-resistant) or progressed during treatment (platinum-refractory).

### Patient inclusion criteria

**Figure 1:**
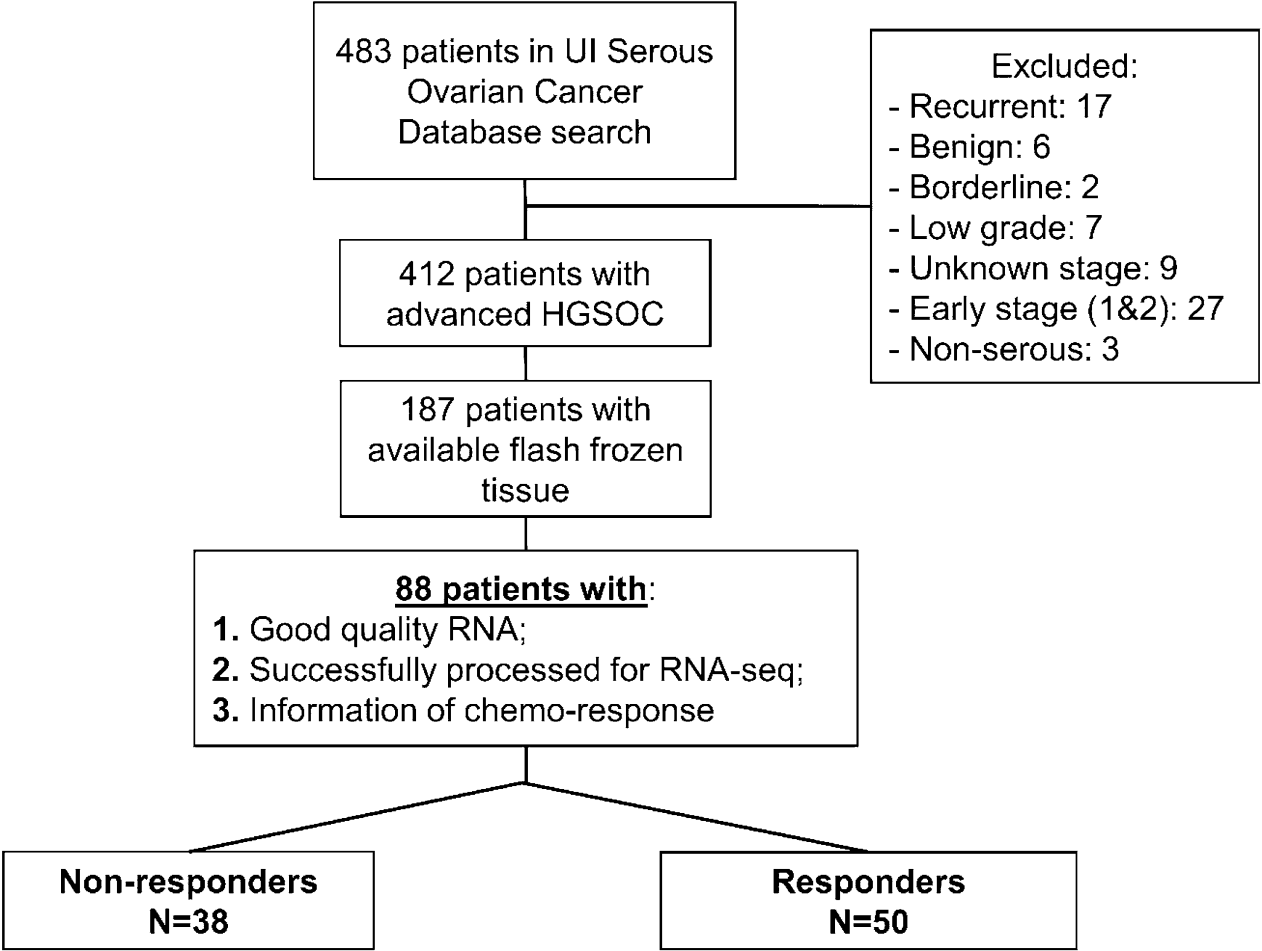
Selection criteria for patients in clinical prediction analysis.

Ovarian cancer patients with high grade serous histology and complete clinical and pathological data were included. Patients that had less than 6 months of follow-up after completing initial chemotherapy, unknown disease status after 6 months of completion of initial chemotherapy, or incomplete data about the chemotherapy delivered were excluded. Also, patients without DNA and RNA of sufficient quality (see below) for RNA-seq or DNA methylation analysis were excluded from the study. Based on the definition of response to treatment, there were a total of 50 patients classified as responders and 38 as non-responders included in the study (Fig. 1). All patients received combination platinum-based chemotherapy initially. However, in 2 patients the regimen was changed before finishing because of disease progression in one case, and stable disease in the other. 66% of patient in our analysis received Taxol as initial treatment.

The institutional review board (IRB) of the University of Iowa (UI) approved the current study including human subjects/materials on April 25, 2018 (IRB Number 201804817: ‘*Prediction Models in Ovarian Cancer*’). The UI Department of Obstetrics and Gynecology maintains a Women’s Health Tissue Repository (WHTR) containing more than 60,000 biological samples, including more than 2500 primary gynecologic tumors^28^. All tissues in the WHTR are collected under informed consent of patients in accordance with University of Iowa IRB guidelines (IRB Number 200910784 and IRB Number 200209010). Tumor samples were collected, reviewed by a board-certified pathologist and flash frozen. HGSC diagnosis was confirm in paraffin. Specimens had less than 30% of necrosis.

### Clinical data

Clinical and pathological data were collected from the electronic medical record. Clinical variables previously observed to be associated with treatment response were included in the data collection^27^. Only baseline clinical and pathological characteristics that can be obtained before starting initial chemotherapy were collected. Table 1 shows the main clinical variables collected for the study. Differences between clinical variables between responders and non-responders were assessed with logistic regression. P-values < 0.05 were considered statistically significant. All clinical variables initially used in the analysis are described in Supplementary Methods. Statistical analysis and graphics were performed with R statistical package and computer environment^29^.

**Table 1:** Patient characteristics and association with treatment response.

			Responders	Non-responders	p-value
			N = 50	N = 38	
Age (median, range)			56 (25–81)	64 (33–83)	0.009
Charlson Comorbidity Index*	1–3		9	6	0.039
	4–6		35	21	
	> 6		1	6	
	Unknown		5	5	
FIGO stage	3		39	25	0.069
	4		7	12	
	Unknown		4	1	
Disease in upper abdomen (other than omentum) by imaging	Yes	Large bowel (N = 4)	28	29	0.051
		Porta—hepatis (N = 4)			
		Mesenteric mets (N = 4)			
		Other (N = 22)			
	No		22	9	
Disease in the chest by imaging	Yes	Chest (N = 4)	6	0	0.991
		Pleural effusion (N = 5)			
	No		44	38	
Grade	2		8	11	0.146
	3		35	23	
	Unknown		7	4	
Residual disease after surgery	Microscopic		12	3	0.053
	Macroscopic		37	35	
	Unknown		1	0	
	Optimal (< 1 cm)		37	20	0.039
	Suboptimal (≥ 1 cm)		13	18	
Removal of pelvic LN	Yes		9	4	0.333
	No		41	34	
Removal of para-aortic LN	Yes		5	3	0.734
	No		45	35	
Surgical complexity score^#^	Low		22	23	0.990
	Intermediate		28	12	
	High		0	3	
Neoadjuvant chemotherapy	Yes		2	10	0.008
	No		47	28	
	Unknown		1	0	
Number of cycles delivered	< 6		2	8	0.344
	≥ 6		48	30	
Dose dense chemotherapy^+^	Yes		1	1	0.844
	No		49	37	

*Charlson Comorbidity Index is a measure of the prognostic burden of all associated morbidities to predict mortality, and is the most validated measure of the prognostic impact of multiple chronic illnesses (www.charlsoncomorbidity.com).

^#^Surgical complexity score: score to predict surgical morbidity and 90-day mortality after primary debulking surgery for HGSC^67^.

^+^Dose dense chemotherapy: increases the dose intensity of the regimen. In serous ovarian cancer, dose dense therapy consists in increasing IV administration of paclitaxel from every 3 weeks to weekly.

### Biological data

#### RNA purification and sequencing

Of the 187 patients identified in the original HGSC panel, 88 primary tumor tissues with sufficient RNA yield and quality were available for analysis; 50 were responders and 38 non-responders (Fig. 1). Most tumors were collected from the ovaries, 63%; 30% were extracted from the omentum, 3% from a pelvic mass and the rest, 4%, from an abdominal mass. The were no differences between both groups (p = 0.2), responders and non-responders, in this distribution. At the time of diagnosis, these HGSC were considered of ovarian origin. Now we assume they would be tubal. Only 3 of them had no ovaries (previously removed) and were considered as primary peritoneal.

Total cellular RNA was purified from primary tumor tissue using the mirVana (Thermo Fisher, Waltham, USA) RNA purification kit following the manufacturers’ instructions. Yield and quality of purified cellular RNA was assessed using a Trinean DropSense 16 spectrophotometer and an Agilent Model 2100 bioanalyzer. Samples with an RNA integrity number (RIN)^30^ greater than or equal to 7.0 were selected for RNA sequencing.

RNA processing and sequencing has been described elsewhere^31^. Briefly, equal mass total RNA (500 ng) was quantified by Qubit measurement (Thermo Fisher, Waltham, USA). Each qualifying tumor was fragmented, converted to cDNA and ligated to bar-coded sequencing adaptors using Illumina TriSeq stranded total RNA library preparation (Illumina, San Diego, CA, USA). Molar concentrations of the indexed libraries were confirmed on the Agilent Model 2100 bioanalyzer and libraries were then combined into equimolar pools for sequencing. The concentration of the pools was confirmed using the Illumina Library Quantification Kit (KAPA Biosystems, Wilmington, MA, USA). Sequencing was then carried out on the Illumina HiSeq 4000 genome sequencing platform using 150 bp paired-end SBS chemistry. All library preparation and sequencing were performed in the Genome Facility of the University of Iowa Institute of Human Genetics (IIHG). Quality control (QC) of both DNA methylation arrays and RNA-seq experiments were performed to minimalize technical biases (see details in Supplementary Methods).

#### DNA methylation assay

Genomic DNAs (gDNAs) were purified from frozen tumor tissues using the DNeasy Blood and Tissue Kit according to manufacturer’s (QIAGEN) recommendations. Yield and purity were assessed on a NanoDrop Model 2000 spectrophotometer and used a 260 nm/280 nm absorbance ratio of ~ 1.8 with minimal to no degradation as shown through horizontal agarose gel eletrophoresis. For more details please see the original publication of DNA methylation assessment in HGSC^32^. Bisulfite converted gDNAs from HGSC tumors were submitted to the Genomics Core Facility of the IIHG for processing on methylationEPIC arrays. The Illumina Infinium MethylationEPIC BeadChip Kit (Illumina, San Diego, CA, USA) allows quantification of more than 850,000 methylation sites across the human genome. Bisulfite-converted samples were denatured and neutralized before they were isothermally amplified overnight. The amplified product was fragmented enzymatically. After isopropanol precipitation, fragmented DNA was resuspended and placed onto Illumina methylationEPIC BeadChip and hybridized. There are two different bead types for each CpG locus, representing methylated or unmethylated DNA. The BeadChip was washed to remove unhybridized DNA, followed by extension and staining. The arrays were scanned with the Illumina iScan and methylation intensity measured. Analysis was performed using the Minfi R statistical package^33^.

#### Pre-processing of biological data

RNA-seq reads were mapped and aligned to the human reference genome (version hg38) using STAR, a paired-end enabled algorithm^34^. BAM files were produced after alignment. We used featureCount to measure gene expression from BAM files^35^. After the gene counts were generated, we used DESeq2 package to import, normalize and prepare data for analysis^36^. We independently used gene expression and micro RNA (miRNA) expression for the association analysis. Exon specific expression needed different mapping references for alignment, therefore ENSEMBL was used to annotate single exons during the mapping process. Then, single exon features were extracted from these newly created BAM files with the DEXSeq package^37^. BAM files for each sample were also used for genomic or single nucleotide variation (SNV) discovery and base-calling against the human genome reference utilizing SAMtools and BCFtools for sorting and indexing^38^. After filtering for duplicates, known non-synonymous single-nucleotide variants, and synonymous variants, results were annotated with ANNOVAR and formatted to display the number of variants per gene and sample^39^. We included only non-synonymous variants. To estimate gene copy we used SAMtools and CopywriteR using BAM files as input^40^. CopywriteR is a suite of tools that uses off-target sequenced data to detect CNV and, initially, was conceived to be used with DNA sequencing products. However, due to the particularities of the method, that uses off-target (not exonic) reads uniformly distributed along the genome, which also are available even in low-coverage sequencing, we used this method to create variables that would be proxies for gene copy in the prediction model (gene copy estimation, or GCE). CopywriteR software uses the segmentation algorithm CBS (circular binary segmentation) to create segmentation files that contain log2-transformed, normalized ratios of read counts, that can be used to do further prediction analyses. Long non-coding RNA (lncRNA) were determined using BAM files as input^41^. Fusion-genes were determined from *fastq* files processed with the STAR-Fusion suite^42^. Supplementary Fig. S3 depicts the pipeline and analytics used for file pre-processing before modelling.

### Statistical analysis

#### Variable selection for prediction modeling

In the prediction model, we only used those variables that could be assessed at baseline, prior to initiation of treatment. RNA features were used only to create predictions models of response to treatment, not for other comparisons. Most RNA features were used as continuous variables. Only presence and absence of SNV and fusion-genes were used as dichotomous variables: present or not. Our approach was to (1) reduce the number of variables using a univariate selection of prediction variables with cross-validation; (2) utilize those significant variables from the univariate selection process in a multivariate model to predict response risk. Rather than introducing all variables directly in the prediction model, this approach was chosen because it would likely lead to a model with less complexity (i.e., fewer variables) and can be more easily validated retrospectively and prospectively. To reduce the number of variables, initially, we introduced only features that were different for both groups in a univariate analysis with ANOVA (p-value < 0.05). Then, cross-validation with 10 replicates for each fold (tenfold) was applied to select those variables that were more informative for prediction of response, as implemented by the *caret* R package^43^. Features that were selected by this univariate analysis were then used for multivariate *lasso* regression modeling. Unless resampling is included in this initial feature selection step, cross-validation of the subsequent models could be biased^44^. Thus, variable selection for all classes of clinical and biological data (gene, miRNA, exon and lncRNA expressions, GCE, fusion-gene, genomic variation, and DNA methylation analysis) was performed with cross-validation to decrease the possibility of overfitting the final model^43^. As result of this selection process, poorly annotated features, present in one or few samples, were eliminated early in the analysis.

#### Prediction model construction

Selected clinical and types of molecular variables from the k-fold cross-validation process were analyzed individually and in combination to determine their prediction potential for treatment response. The lasso method, as implemented in the glmnet R package^45^, was used to develop a regression model to predict responders versus non-responders. We selected lasso because it is a multivariate regression method that allows simultaneous selection and estimation of the effects of variables, while accounting and adjusting for confounding factors. In our experience, lasso consistently lowers number of samples and computes AUC without reporting any errors, as compared to other prediction methods^22^. We evaluated the performance of our model using the AUC and its 95% confidence interval (CI). AUC was estimated with 1,000 replicates of tenfold cross-validation to avoid over-fitting of the model (internal validation)^46^. Bias-corrected and accelerated bootstrap CIs were computed for resulting AUCs. A value of 0.5 indicates a lack of model predictive performance, and 1.0 indicates perfect predictive performance, or the best model.

#### Model validation

For external validation of response prediction models created with UI data, we used a publicly available TCGA HGSC dataset^47^. We included only patients with follow-up of at least 6 months after completing initial chemotherapy, with known disease status after 6 months of completion of initial chemotherapy, and data about type of chemotherapy delivered^25^. As before, only baseline (pre-treatment) *clinical data* were used for validation (see Supplementary Methods). Not all clinical data used in the UI cohort was available in TCGA. This is a limitation of using TCGA data for model building^27^. Based on these inclusion criteria, there were a total of 189 patients classified as responders and 149 as non-responders in TCGA for validation of prediction models (Supplementary Table S2).

To make validation possible, only TCGA patients with RNA-seq HGSC tumors were included in the analysis. BAM files, resulting from RNA-seq alignment to hg38 human genome version were downloaded (DbGaP Access #16,003—NCBI) and pre-processed as for the UI dataset (see *Pre-processing of biological data*). Fusion-genes need to be determined from *fastq/fq* files, so we converted BAM files into *fq* files with BCFtools^38^. After sorting and indexing, *fq* files were processed with the STAR-Fusion suite to obtain fusion-genes^42^.

The *validation analysis* applies the UI-built model to the TCGA data to predict or discriminate between responder or non-responder classes. For validation of clinical data, we constructed new UI models with clinical variables available in TCGA. The same was done for other types of data with missing variables in TCGA dataset, DNA methylation and fusion-genes. For validation, we used the best UI-built models of treatment response. Next, we used the R package *pROC* to determine thresholds, or cut-offs, for the UI-built model applied to the TCGA data (see details in Supplementary Methods)^48^. Threshold values that yielded sensitivities of > 90% were ranked from highest to lowest sensitivity, negative predictive value and AUC. Among the ranked results, the top-ranked set of tuning parameters was used to fit a final score of the model to the entire set of patients and define the classification rule. A sensitivity threshold of over 90% will identify most of the patients at risk of failing treatment. Our goal is to capture the highest proportion of non-responders for clinical use of the model, while also aiming for acceptable specificity. Similar thresholds have been used to assess tests for malignancy, recurrence or failure of ovarian cancer treatment^49–53^. We coupled high sensitivity with high accuracy measured by AUC: 0.8–0.9 is considered ‘a very good’ diagnostic accuracy, 0.9–1 is considered ‘excellent.’

In previous studies we observed that TCGA patient population has different genetic admixture than UI patient population^54^. That difference may influence the performance of validation analyses. To account and adjust for those genetic differences we extracted genotypes from VCF files obtained after RNA-seq. Then we employed two different strategies for the adjustment: 1) we performed a principal component analysis (PCA) to differentiate genotypes from UI and TCGA datasets and used the first 3 principal components (PC) for adjustment; 2) we performed a lasso regression analysis (PCA) to obtain the genotypes that differentiated UI from TCGA, and used them for adjustment (for details see Supplementary Methods).

#### Survival analysis

To assess the association of survival with response, survival analysis was performed using Cox proportional hazard ratios.**

### Ethical approval and consent to participate

Tumor samples were obtained under informed consent after approval by the University of Iowa Institutional Review Board: IRB# 201,804,817 (approved 5/9/2018) and 200,209,010 (approved 9/19/2005). The institutional review board (IRB) of the University of Iowa (UI) approved the current study including human subjects/materials on April 25, 2018 (IRB Number 201804817: ‘*Prediction Models in Ovarian Cancer*’). All data collection and processing, including the consenting process, were performed after approval by the University of Iowa IRB.

### Consent for publication

All authors have reviewed and approved the manuscript for submission.

## Results

In the UI database, 43% of patients were non-responders, and in TCGA HGSC dataset 44% of patients were non-responders, chi-square p-value = 0.88 (Table 1 and Supplementary Table S1, respectively). Non-responder UI patients had higher Charlson comorbidity index score, more residual disease after surgery, and received more frequently neoadjuvant chemotherapy before surgery (Table 1). Non-responder TCGA patients had more residual disease after surgery (Supplementary Table S2). Median survival was 39.3 months (95% CI: 31, 58.2) for UI responders and 57.7 months (95% CI: 44.3, 82) for TCGA responders. Median survival was 12.5 months (95% CI: 8, 19.1) for UI non-responders and 22.7 months (95% CI: 15.9, 26.3) for TCGA non-responders. Based on these 95% CIs, there were no differences in survival for responders and non-responders in the UI and TCGA datasets.

### Variable selection for prediction modeling

**Figure 2:**
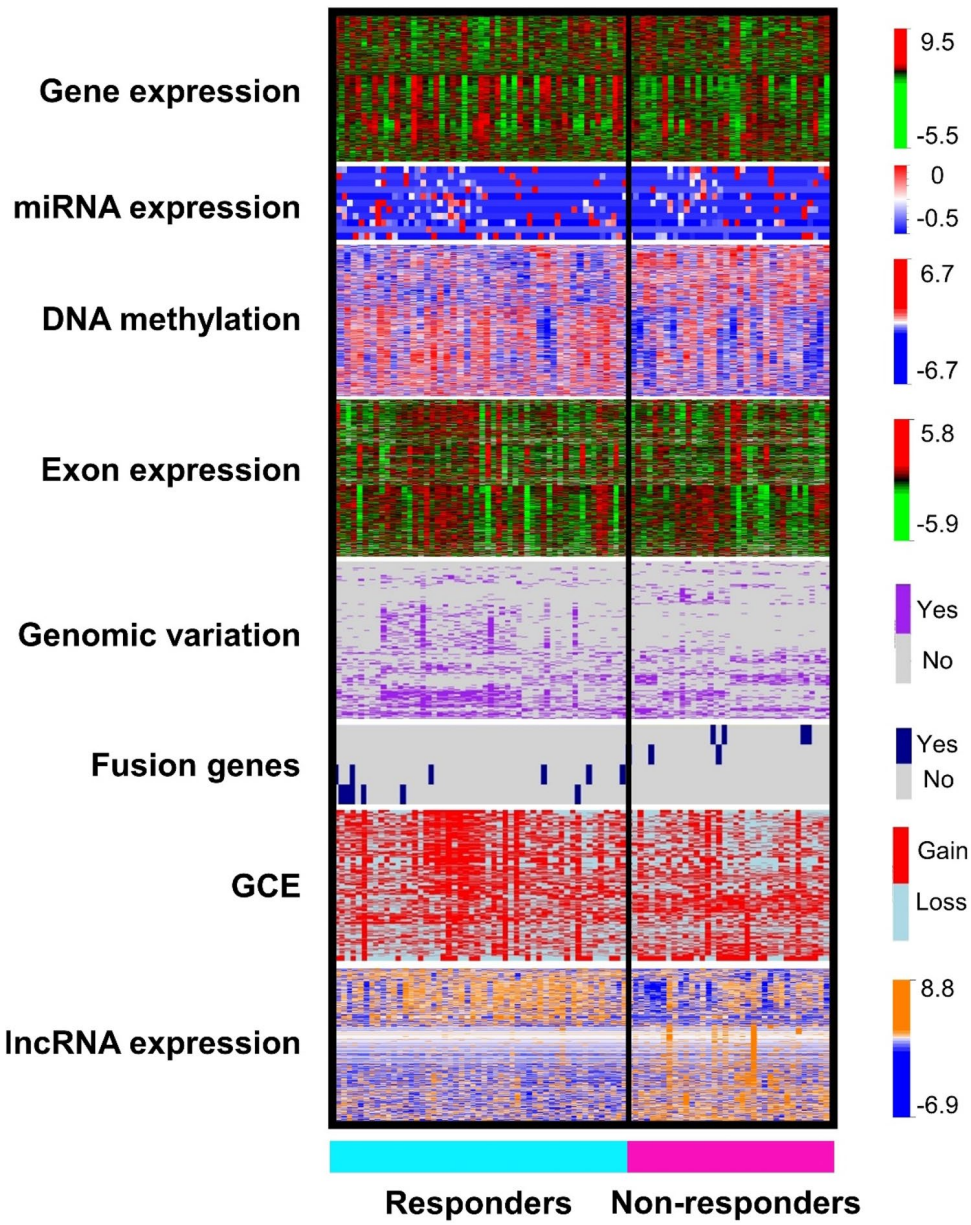
Heatmap of selected variables after univariate ANOVA analysis. Representation of the significant variables after univariate analysis (p < 0.05) for different types of genomic data: gene, miRNA, exon, and long non-coding RNA (lncRNA) expression, DNA methylation, genomic variation, fusion-gene presence, and gene copy estimation (GCE). At the right side of each heatmap there are color-coded range of values for all genomic variables. Heatmaps were generated with R package *Heatplus*^68^ (R version 3.6.3. http://www.r-project.org).

After the univariate analysis of all clinical and genomic data with ANOVA as described in Methods, we identified those variables that were more informative for the outcome of interest: treatment response (Fig. 2). The number clinical, gene, miRNA, exon and lncRNA expressions, GCE, fusion-gene presence, SNV, and DNA methylation variables selected after the univariate and multivariate analysis, and included in the prediction analyses are detailed in Table 2. Notably, in the genomic variation analysis, we found BRCA2 variants in 24% (12 out of 50) of responders and in 26% (10 out of 38) of non-responders (p = 0.52); and BRCA1 variants in 36% (18 out of 50) of responders and 50% (19 out of 38) in non-responders (p = 0.06). These differences were non-significant; therefore BRCA1&2 were not selected for the prediction analysis.

**Table 2:** Variable selection and variables after prediction model construction with type of data.

Type of data	Initial number of variables	Variables after selection: univariable ANOVA analysis with k-fold cross-validation	Variables after multivariable prediction model with lasso
Clinical	45	–	7
Gene expression: mRNA	23,528	2214	62
miRNA expression	1914	12	11
Gene copy estimation: GCE	28,917	1098	83
Genomic variation	13,840	327	54
DNA methylation	66,042	4961	35
Long non-coding RNA	16,325	773	69
Fusion genes	597	147	104
Individual exon expression	63,677	4387	61

To reduce the number of variables, we used univariate analysis of all data with ANOVA to select the variables that were more informative for prediction of response, with a p-value < 0.05 (3rd column). Features that were statistically significant in this univariate analysis were then used for multivariate *lasso* regression modeling. In the last column are the number of variables resulting after performing that prediction model with only one variable. Variables in this last column were used to build prediction models integrating 2 or 3 types of data.

### Prediction model construction

**Figure 3:**
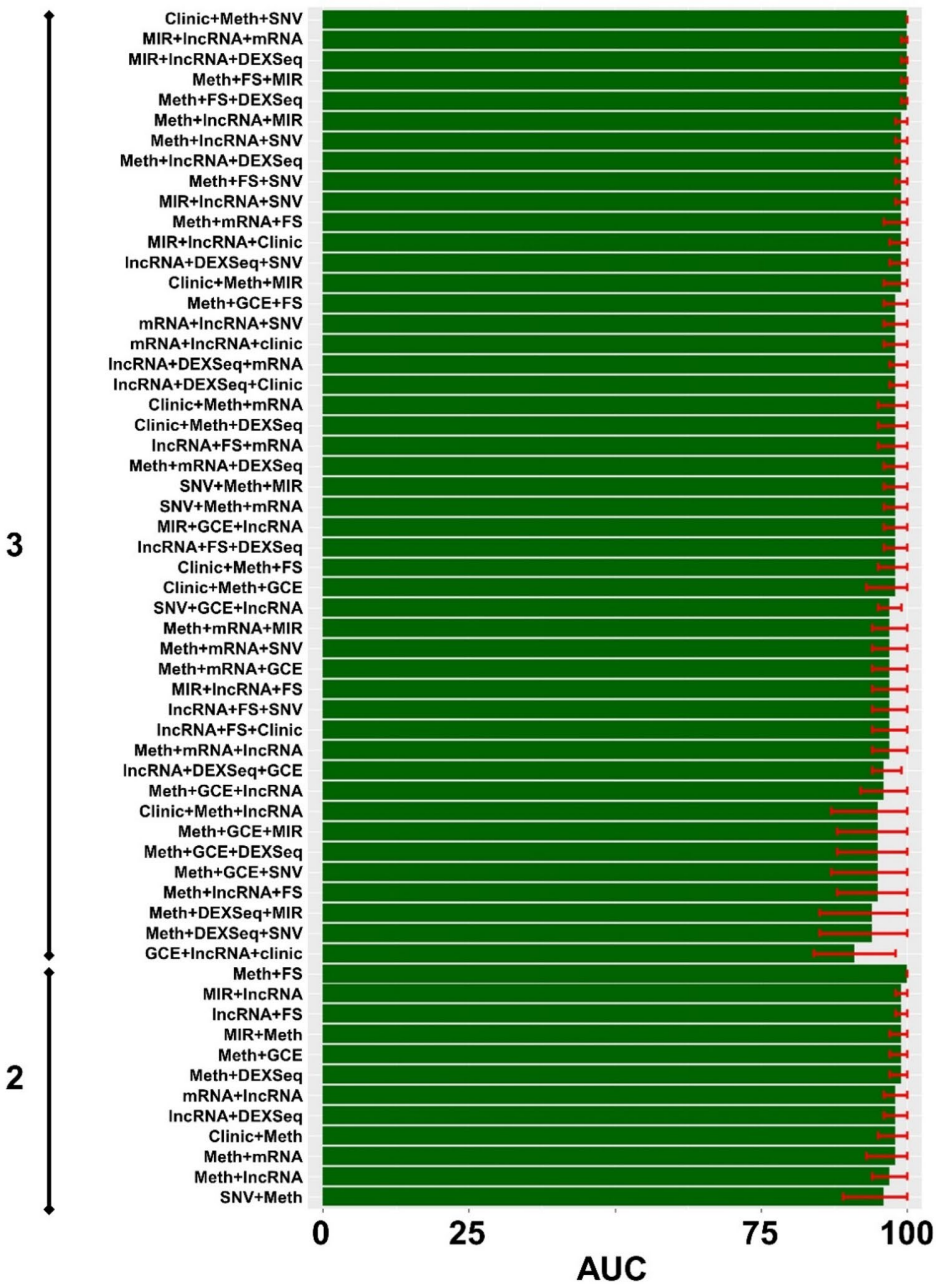
High performing prediction models of response. On the left is the number of types of data: **2**: combination of 2 types of data; **3**: combination of 3 types of data. Different performances are displayed in ascending order. The x axis is AUC as a percentage (0–100%). Although we tested 107 models with 1, 2 and 3 variables, we only represented those models with performances over 90% measured in AUC, N = 59 (to see all of them see Supplementary Figure S7). FS: Fusion genes; Meth: DNA methylation; SNV: single nucleotide variation; GCE: gene copy estimation; DEXSeq: exon expression; lncRNA: long non-coding RNA; MIR: micro RNA, mRNA: gene expression. Graphics were generated with R package *ggplot* (R version 3.6.3. http://www.r-project.org)^69^.

Prediction models of response were built initially with one type of selected data: clinical, gene, exon, miRNA, and lncRNA expression, SNV, GCE, fusion-gene presence, or DNA methylation (Table 2). See Supplementary Table S3 for more details about the variables after lasso prediction. Next, we built models integrating 2 and 3 types of data. The performance of all models was evaluated using the AUC and its 95% CI. By integrating clinical and genomic variables, we achieved prediction performances of over 95%. Figure 3 represents all prediction models with 1, 2, or 3 variables with AUC over 90% (N = 59). Adding 4 or more types of data increased model complexity without a significant improvement in performance. For details about all prediction models review Supplementary Figure S7.

### Model validation

**Figure 4:**
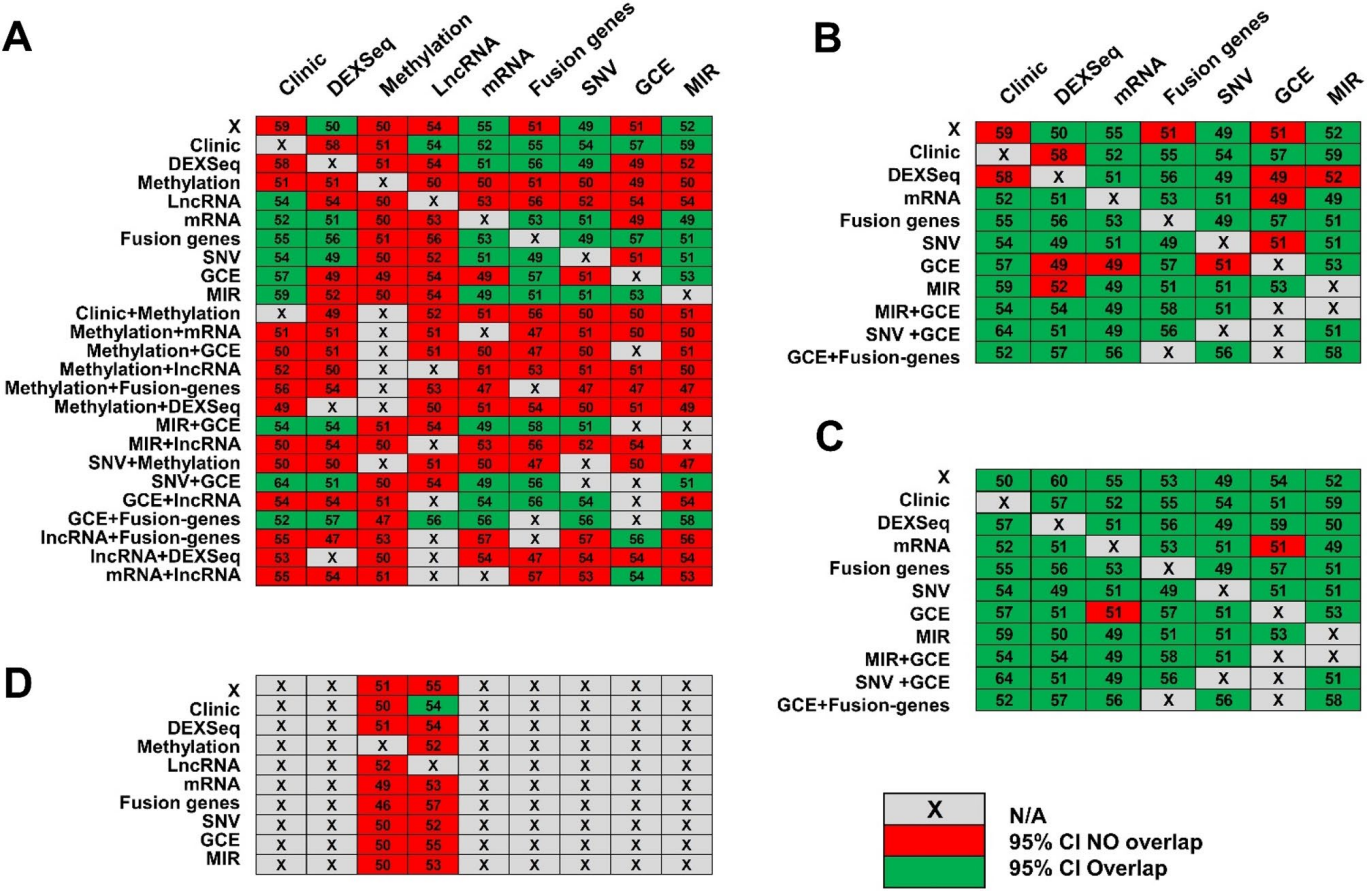
Validation of UI prediction models of response to treatment in TCGA datasets. The columns represent different types of clinical and molecular data: DEXSeq (individual exon expression), Methylation (DNA methylation), LncRNA (long non-coding RNA expression), mRNA (gene expression), Fusion genes (presence of fusion genes), SNV (Single Nucleotide Variation), GCE (gene copy estimation), and MIR (microRNA expression). The rows also represent different types of data, either individually or in combination. (**A**) Validation of all prediction models of response in TCGA: Models containing DNA methylation and lncRNA data underperformed: AUC 95% CIs of TCGA-validation models did not overlap with AUC 95% CIs of UI-built training models (red cells in the graphic). Green cells represent those AUC 95% CIs of TCGA-validation models that overlap with UI-built AUC 95% CIs. (**B**) When we removed models containing DNA methylation and lncRNA data, 80% of CIs from UI-built training models overlapped with CIs from TCGA-validation models. (**C**) When prediction models without DNA methylation and lncRNA data were adjusted with genotyping data (as detailed in in Supplementary Methods), 97% of CIs from UI-built models overlap ped with CIs of TCGA-validation models. (**D**) TCGA-validation performance did not improve when adjusting for different genotypes between UI and TCGA. This was tested in models with 1 and 2 types of data (18 models).

One of the limitations of using TCGA for validation of prediction models was that not all clinical data used in the UI cohort were available in TCGA dataset. For validation analysis, we took all models built using 1, 2, or 3 variables in the UI dataset and inserted TCGA data to assess how well the UI-build models discriminate between responders and non-responders in the TCGA dataset (107 different models)^47^. We selected a sensitivity threshold over 90% in order to identify most of the patients at risk of failing treatment (see the rationale in methods). Notably, validation of models containing DNA methylation and lncRNA data underperformed (Fig. 4A). If we eliminated models with DNA methylation and lncRNA, 80% (51 out of 64) of UI-built models had an AUC 95% CI in the TCGA validation set that overlapped with the UI training set interval (Fig. 4B).

To adjust for different population genetic backgrounds between UI and TCGA cohorts, we used PCA and lasso regression analysis with genotypes that differentiated UI from TCGA. The preliminary studies with PCA did not increase validation performances (see Supplementary Methods), so we next carried out the adjustment with genotypes that differentiated UI and TCGA datasets. When adjusting for genetic variation, validation of the UI-built models had an AUC 95% CI in the TCGA validation that overlapped 97% (62 out of 64 models) with the UI training set interval (Fig. 4C). We did not observe any improvement after adjusting for genotypes in models containing DNA methylation and lncRNA data (Fig. 4D). These results indicate that differences in performance between UI-built and TCGA validation models that do not contain DNA methylation and/or lncRNA data may be related to different genetic background of both datasets.

## Discussion

Prediction of treatment response in HGSC patients before treatment initiation is a difficult task. Not many groups have attempted these types of predictions and, of the few reported models, none have been introduced successfully into clinical practice. The reasons are varied but can be classified into two groups: (1) prediction model performances did not warrant their introduction in routine practice^2,13^; or (2) models could not be validated in independent datasets and thus could not be generally applied^14,21^. We present a comprehensive study of treatment response prediction in patients with advanced stage HGSC. Most importantly, these prediction models were validated in an independent dataset with similar in-depth genomic assessment, TCGA.

Prediction models were built by integrating different types of clinical and molecular data. Simpler models were built after selecting those variables more informative for the outcome, treatment response, and using cross-validation to minimize over-fitting. As we increased model complexity (with 2–3 types of data), performances on the training set reached levels over 95% in terms of AUC. These performances are promising and could provide robust clinical decision support to discriminate responders versus non-responders. The most predictive models included diverse types of data, but notably, those around 100% AUC included epigenetic regulators of gene expression, either by DNA methylation or miRNA expression. Also, other gene modulators like lncRNA expression and individual exon expression, a readout for splice variant expression^55^, were involved in these high performing models. Finally, clinical information was also an important component of the best prediction models. Other components of high performing models, like fusion genes, have not been characterized yet in HGSC outcome prediction or prognosis, but have been associated with acquired resistance^56^.

Epigenetic gene regulation is one of the mechanisms that regulate treatment response. For example, whole genome DNA methylation analysis found that epigenetic regulation of potentially clinically relevant genes predicts response to platinum^57–59^. Also, lncRNAs have been associated with epigenetic regulation of HGSC^60^, and specific lncRNAs have been associated with chemo-resistance^61^. Therefore, it is not surprising that some of the best prediction models are composed of diverse lncRNAs. The role of miRNAs in response to treatment in HGSC in vitro is well documented^62^, and some miRNAs also have been associated with chemo-response modulation^63^. The presence of epigenetic regulators and modifiers of treatment response in high performance prediction models of response to therapy seems to support these regulatory mechanisms. We must be cautious about extrapolating functional conclusions from prediction models. Prediction analysis is a form of statistical learning that uses data obtained in the past to predict outcomes, or behavior, of other individuals in the future. Prediction analysis is based on association and does not infer causation^64^.

Prediction models of response created with UI data were validated in TCGA HGSC data. Notably, models containing DNA methylation and/or lncRNA data did not perform well in validation analyses. Moreover, when we adjusted for different genetic backgrounds between UI and TCGA samples, validation performances of models *not* containing DNA methylation or lncRNA improved, with 97% of overlap in the 95% CI of AUCs. Conversely, validation models containing DNA methylation and/or lncRNA did not improve despite adjusting for genetic background. We speculate that genetic background differences between the UI-sampled population and TCGA account for some variation in the validation of these prediction models of treatment response, except for those models including DNA methylation and/or lncRNA. The poor performance of validation TCGA prediction models including DNA methylation and/or lncRNA data was likely due to other reasons. DNA methylation analysis for TCGA HGSC data was performed using Illumina Infinium HumanMethylation27K BeadChip arrays^47^. Methylation in UI was performed with an EPIC BeadChip 850 k arrays (both arrays from Illumina Inc.). The 27 K methylation array interrogates mainly CpG islands in gene promoter regions, while the 850 K array also explores DNA methylation outside the promoter areas with whole genome coverage^32^. To determine lncRNA expression in UI HGSC data, we used data from RNA sequencing that was carried out on the Illumina HiSeq 4000 genome sequencing platform using 150 bp paired-end sequencing by synthesis (SBS) chemistry^32^. LncRNA expression was extracted from TCGA HGSC sequenced with the Illumina HiSeq 2000 genome sequencing platform that uses 75 bp paired-end SBS chemistry. Differences between 150 and 75 bp sequencing products may have contributed to differences in background noise and total lncRNA counts. Indeed, differences between the platforms may contribute to the decrease in performance validation with lncRNA data. Other technical differences between both databases, like libraries preparations between both sets, may influence critically overall prediction model performance.

A strength of this study is that we used diverse databases of genomic and clinical variables to build prediction models of response. We postulated that a complete database containing all variables involved in malignant cell functions would make prediction models more accurate^22,27,31,65^. Therefore, we extracted as much information from the HGSC specimens as possible to improve our models. Likewise, with clinical data we extracted as much baseline clinical information that could affect the primary outcome of interest. These variables may have been known previously to affect the outcome, or not. Public databases not designed specifically for prediction assessment, like TCGA, may lack of some characteristics that result in important discrepancies of model performance. In the present study, we were able to adjust for these discrepancies in some of the models, except for those containing DNA methylation and/or lncRNA data. Another strength is the outcome definition. Progression-free survival of at least 6 months after the first platinum-based treatment, or responders, is an standard definition of response to chemotherapy^2,5,6^. Indeed, patients that do not respond to initial standard treatment, platinum-resistant, or progressed during treatment, platinum-refractory, are considered a different population when chemotherapy or clinical trials are considered^4,8^. Treatment response was reviewed in all UI patients, and any patient that did not meet the inclusion criteria was excluded from the analysis. Also, to be included in validation analyses all TCGA patients had to meet treatment response definition criteria. Finally, validation of all models of prediction in a public, well known, independent database (TCGA) also strengthens our study. One of the inherent limitations of the study comes from its design: to build prediction models, initially, the outcome must be known, so the initial step of the analysis and validation is retrospective. The advantage of extracting data from patients of a single institution is the uniformity of diagnosis, outcome definition, specimen collection and processing, treatment philosophy and surveillance. This resulted in a homogenous population with quality biological and clinical data. The selection process may have some disadvantages, though, and the selected samples may lack diversity and it may be limited by the number of HGSC eligible patients. We are the largest of only two Gynecological Oncologic practices that serves around 80–90% of women with gynecological cancer in the state of Iowa (USA). Thus, the samples we have studied represents the female racial composition of the State of Iowa: 95.5% white, 1.1% black, 3.4 other (Latina, Asian, Pacific)^54^. We adjusted these differences in genetic variation with TCGA during validation (see Results). In future studies, it may be especially important that every center that treats women with HGSC knows exactly the racial and genetic composition of the population they treat, so they can correct or adjust for these differences. We acknowledge the limited sample size used to construct prediction models, but the necessity of having an accurate outcome definition and homogeneous population is even more critical. Previous prediction studies are plagued with patients with heterogenous clinical characteristics and outcome definitions, and with different histological types of ovarian cancers that made generalizability even more difficult^2,13,14,21^.

Before these models can be applied clinical, they must be validated prospectively. Despite internal and external validation, models of prediction still may have biases due to overfitting. In the prospective model those biases could be detected and corrected before clinical application. Then, for models of prediction to be applied, there is a process that must be followed. After a biopsy is taken (either CT-guided or during surgery) and HGSC histologic type has been confirmed, we will determine all components of the best prediction model validated prospectively. As long as CT-guided biopsies have enough tumor cellularity (over 2/3), and not too much necrosis (< 30%), it would be enough and comparable to the initial model. Sequencing is rapidly evolving with single cell RNA-seq technology^66^. Each of these components will be transformed as they were formatted in the initial analysis (i.e., log transformed, coding values, etc.) and the values would be applied to the weight of each variable in the selected model. The addition of all values will give us a final score. That score, and where it is located with respect to a chosen threshold, will assess the risk of the patient to fail initial therapy. We could design customized assays, PCR-based, for the genomic features of the model that would reduce costs and complexity and would improve the turnaround time so the results could be used even before surgery. With this information, clinicians could have a good sense of which patients would respond to treatment, and they would be better informed to plan initial treatment. For example, if a patient has a higher score for response, the surgeon would take that into consideration to balance the effort in cytoreduction with possible complications. Knowing that the patient may not respond as well to initial treatment may increase the surgical effort to minimize residual disease. Also, if a patient has a lower score for response, we may want to involve them in clinical trials that add targeted treatment to the initial chemotherapy backbone to improve outcomes.

## Conclusions

Based on our results and previous reporting from other prediction studies^22,27,31,65^ we can conclude that our hypothesis holds true: integrating comprehensive clinical and genomic data from patients with HGSC results in accurate and robust prediction models of treatment response. We have described high performance prediction models of response for initial treatment in HGSC. Based on these performances, some of these prediction models could be useful to provide clinical decision support that will differentiate responders to non-responders. Furthermore, these models were validated in an independent, trusted, well known database.

## Supplementary Information

Supplementary Information 1.

Supplementary Information 2.

Supplementary Information 3.

## Data Availability

Data for the prediction model has been submitted to the GEO at NCBI website: https://www.ncbi.nlm.nih.gov/geo/. Datasets with methylation data can be browsed by their accession number: GSE133556. Datasets with RNA-seq can be browsed by their accession number: GSE156699. The validation part of this study was performed in silico, with de-identified publicly available data. All data from TCGA is available at their website: https://portal.gdc.cancer.gov/. Software utilized by this study is also publicly available at Bioconductor website: http://bioconductor.org/.

## Abbreviations

HGSC: High-grade serous ovarian cancer
RNA: Ribonucleic acid
DNA: Deoxyribonucleic acid
lncRNA: Long non-coding RNA
miRNA: Micro RNA
ANOVA: Analysis of variance
AUC: Area under the curve
TCGA: The cancer genome atlas
CI: Confidence interval
PFS: Progression-free survival
OS: Overall survival
IRB: Institutional review board
WHTR: Women’s health tissue repository
gDNAs: Genomic DNAs
SNV: Single nucleotide variation
CNV: Copy number variation
GCE: Gene copy estimation
NCBI: National Center for Biotechnology Information
PCA: Principal component analysis
PC: Principal components
UI: University of Iowa
ROC: Receiver operating characteristic
FIGO: International federation of obstetrics and gynecology
GEO: Gene expression omnibus
FDR: False discovery rate
