# Supplementary material for: Creation and validation of models to predict response to primary treatment in serous ovarian cancer
Source: Sci Rep. 2021 Mar 16;11:5957. doi: 10.1038/s41598-021-85256-9 (PMC7971042; doi:10.1038/s41598-021-85256-9)
Supplement: Supplementary file 1 — Supplementary Information 1. [file 41598_2021_85256_MOESM1_ESM.docx]

**Supplementary Table S3:** Selected variables after ANOVA and initial multivariate lasso analysis.^#^

| Type of Data | Variables after lasso prediction | Variables |
| --- | --- | --- |
| Clinical | 7 | Age, disease in upper abdomen, disease in colon-upper abdomen, disease in porta-hepatis, other organs affected in upper, optimal surgery, neoadjuvant chemotherapy |
| Gene expression: mRNA | 62 | SYNC, CC2D1B, LINC00466, GBP3, CD160, CFHR3, REN, IL20, LINC00467, GCSAML-AS1, ITIH5, USP6NL, OPTN, DCLRE1C, ZNF37A, WNT5B, MMAB, SPG21, ADPGK, MAN2C1, PTPN9, SNUPN, CTSH, GOLGA6L17P, MCTP2, ROGDI, PPP4R1-AS1, ZNF24, CDH20, ATG4D, TMEM205, LGALS7B, PINLYP, NTN5, FCGRT, SIGLEC17P, NLRP7, BCL11A, FIGLA, KCMF1, MROH8, LINC01273, LOC102723780, COL6A4P1, GPR87, RAP2B, LOC101927237, TRIM61, SV2C, PANK3, RANBP17, BTNL9, MCUR1, C2-AS1, RTN4IP1, STK31, ZPBP, AKR1B10, QRFP, XG, GLOD5, TKTL1 |
| miRNA expression | 11 | MIR200B, MIR429, MIR3671, MIR553, MIR6755, MIR381HG, MIR4537, MIR4312, MIR3174, MIR650, MIR7161 |
| Gene copy estimation: GCE | 83 | PKLR, LOC645682, LOC645676, LOC284620, MRPS29P1, MSTO1, MR1, IER5, ESRRG, ACBD5, LOC645084, CALM2P2, LOC645333, TCF7L2, FLJ46361, FAM24B, OR52B6, OLFML1, CALCP, BDNF, API5, CYCSP29, C11orf44, FLJ10652, OR6C3, OR6C7P, ZNF664, SMAD9, FLJ31945, LOC645814, LOC653381, LOC644761, NOPE, IMAA, LOC646828, DREV1, IGSF6, FLJ38101, LOC643911, LOC388279, IRX5, ANKFY1, UBE2G1, MGC29671, KRTHA6, DHX40, PPP4R1, LOC441847, GPR77, LOC474340, ADCY3, SLC20A1, PSCDBP, LOC645406, FLJ39660, C21orf127, HSPDP7, LOC391282, FLJ26056, CRYBB3, CRYBB2, LOC646123, FBLN1, PROM1, PI4K2B, NT5C3P1, IL31RA, NUDT12, C5orf13, MATR3, HK3, LOC442160, RPS10, RSPO3, MESTP1, TWIST1, CRHR2, NBLA04196, LOC646531, TRBV12-4, SLCO5A1, LOC90120, LOC643461 |
| Somatic mutations | 54 | AATK, ADGRE5, ADO, B4GALT2, B9D2, C9orf84, CCDC183, CDC27, CDT1, CHST3, CLDN6, CLEC3B, DLL1, ELP4, FAM24B, FBLIM1, FCGR3B, FCRLB, H2AFY, HR, IFI44L, IL6ST, IPO11, JPH2, KCNMB3, KIAA0141, KIF24, LGALS3, LGMN, LNX2, LSP1, LSS, MCM4, MEDAG, MRPS5, MTG1, MTMR7, MUC20, PCMT1, PLEKHA8, PTGR1, PTPRD, SCO2, SERPING1, SLC38A6, STK32C, SYNE1;SYNE1, TMEM185B, TMF1, WASHC2A, ZDHHC14, ZDHHC8, ZNF268, ZNF727 |
| DNA methylation | 35 | PRDM2*, TMED5, TNRC4*, RASAL2, PTGS2, RGS7, TTC15*, LINC01105*, PDE1A*, ICA1L, ADAM23*, CELSR3, FAM86D, CHST2, C4orf34, SRD5A1, HSPB3*, RAD17, LINC00461*, PCDHA2*, NEDD9*, C6orf211, UNC93A*, ASL, LOC101927870*, KLF14, SLC45A4, NANS, KIAA1217*, PLAC9*, PCGF5, KDM4E*, OR4K5*, SLC26A11*, CPT1C |
| Long non-coding RNA | 69 | SNHG12, AL033527.5, AC105942.1, LINC01357, AC239799.1, AC241644.1, AL590666.4, Z99943.1, AL358176.4, AC007684.1, AC096666.1, AC023469.2, AC009495.1, AC018814.1, SCAANT1, ALDH1L1-AS1, SIAH2-AS1, TM4SF19-AS1, AC010478.1, AC093298.1, AC110011.1, AL590227.1, AL121718.1, LINC01268, AL356124.1, AL353596.1, LINC01625, MAGI2-AS1, AC022182.2, LACTB2-AS1, AC013509.1, MIRLET7A1HG, LINC02635, ANK3-DT, AC025947.1, AC090625.2, AC090692.2, AP001160.4, AC005908.2, AC018653.1, AC010168.2, AC008250.2, AC139768.2, AC023509.3, AC073896.5, FAM222A-AS1, PCOTH, SUCLA2-AS1, AL132780.1, BMF-AS1, AC090825.1, AC092138.1, AC018557.2, AC009061.2, AC015853.2, AC090774.2, SEPT4-AS1, AP005271.1, AC023043.4, AC018445.1, AC005523.1, AC025811.1, AC092295.1, AL135937.1, AJ011932.1, AL133456.1, AL031587.2, AL021707.4, Z68871.1 |
| Fusion genes | 104 | AC004475.1--PRPF6, AC006427.2--TAPT1-AS1, AC007952.4--RMRP, AC011586.2--MSR1, AC060766.7--SLFN12, AC092745.5--AC092745.2, AC104850.2--CX3CR1, AC244035.1--RHEX, ACER2--ACO1, AF235103.3--ZNF250, AL035409.1--ST6GALNAC5, AL353138.1--PTCHD4, AL445985.1--SPATA13, ANKRD28--METTL6, ARL17A--KANSL1, ARMC7--TRIM80P, AUTS2--INO80C, AZIN1--NOL4L, BOP1--PRPF19P1, BTBD10--TEAD1, CCNI--GINS3, CEBPZ--HEATR5B, CHD7--AC097374.1, CHSY1--PCSK6, DLG2--DISC1FP1, DRG1--LIMK2, EED--INTS4, EHBP1L1--CAMKV, EIPR1--FMNL2, ESR1--AKAP12, FAM98B--FRMD5, FGFR2--RHOBTB1, GNB1--CFAP74, HNRNPU--ADSS, IER3IP1--SKOR2, ITGB4--MYO15B, JAM2--MPPED2, JMJD1C--CCNYL1, LINC00958--AC084859.1, LINC01315--TCF20, LINC02408--CAND1, LSM14A--WTIP, LUC7L--AXIN1, MAGED2--ZFAT, MARK3--NDUFB4P11, MECOM--AC116337.3, MVB12A--AP1M2, NCEH1--LPP, NECAB1--SLC26A7, NEK2--LPGAT1, NFE2L1--PNPO, NFKBIB--TEAD1, NOP58--BMPR2, NRIP1--AJ009632.2, NUCB1--ARFGAP3, PACS1--HAUS3, PARP4--BAGE2, PFKFB3--LINC02649, PGM2L1--POLD3, PHACTR4--RPP14, PKP3--METTL15P1, PLEKHG1--PPP1R14C, PPFIBP1--AL591242.1, PRSS42P--PRSS50, PSPC1--ZMYM5, RAB3GAP1--R3HDM1, RABEP1--MINK1, RERE--TNFRSF9, RMND1--ARMT1, RN7SL1--SNORD3A, RORA--ANXA2, RPPH1--WDR74, SCAF8--TIAM2, SIPA1L3--FP236383.1, SLC35D2--ZNF367, SMARCA4--ZNF700, SMBD1P--TFRC, SMIM14--UBE2K, SNORD3A--RPPH1, SPON1--FAR1, SRGAP3--AC068631.1, SSBP1--MGAM, TFDP2--GK5, TMCC1--CD96, TMEM87B--MERTK, TMPO--APAF1, TNFRSF1A--LPCAT3, TNS3--TUSC3, TOGARAM1--FANCM, TPX2--BCL2L1, TRAPPC3--MAP7D1, UBA2--RAD51B, UBE2F--LRRFIP1, UBE2K--SMIM14, USP22--RN7SL2, USP54--ASCC1, VRK2--SPTBN1, ZBTB2--FBLN2, ZBTB8OS--AC090627.1, ZCCHC4--PI4K2B, ZNF274--AC018359.1, ZNF468--PGLYRP2, ZNF609--SNX1, ZSWIM7--AC005747.1 |
| Individual exon expression | 61 | ENSG00000206817, ENSG00000160766, ENSG00000212161, ENSG00000122863, ENSG00000201548, ENSG00000235100, ENSG00000213574, ENSG00000197601, ENSG00000133818, ENSG00000269570, ENSG00000215009, ENSG00000249388, ENSG00000084110, ENSG00000256783, ENSG00000129535, ENSG00000139899, ENSG00000258423, ENSG00000259482, ENSG00000103647, ENSG00000272887, ENSG00000048462, ENSG00000260818, ENSG00000241334, ENSG00000197445, ENSG00000184860, ENSG00000203483, ENSG00000264148, ENSG00000108671, ENSG00000186395, ENSG00000251964, ENSG00000244527, ENSG00000266148, ENSG00000213290, ENSG00000266916, ENSG00000105427, ENSG00000161618, ENSG00000225942, ENSG00000213594, ENSG00000068878, ENSG00000230696, ENSG00000231766, ENSG00000223923, ENSG00000263681, ENSG00000252020, ENSG00000272720, ENSG00000252796, ENSG00000248150, ENSG00000247121, ENSG00000270666, ENSG00000199851, ENSG00000220563, ENSG00000205584, ENSG00000238997, ENSG00000221401, ENSG00000254104, ENSG00000253690, ENSG00000264969, ENSG00000182021, ENSG00000196632, ENSG00000186376, ENSG00000229829 |

^#^To reduce the number of variables, we used univariate analysis of all data with ANOVA to select the variables that were more informative for prediction of chemo-response, with a p-value<0.05. Features that were statistically significant in this univariate analysis were used for multivariate *lasso* regression modeling. Variables resulting after performing that prediction model with only one variable were used to build prediction models integrating 2 or 3 types of data.

*DNA methylation probes located close to a gene or lncRNA, but not in a promoter CpG island.
