# Supplementary material for: Creation and validation of models to predict response to primary treatment in serous ovarian cancer
Source: Sci Rep. 2021 Mar 16;11:5957. doi: 10.1038/s41598-021-85256-9 (PMC7971042; doi:10.1038/s41598-021-85256-9)
Supplement: Supplementary file 2 — Supplementary Information 2. [file 41598_2021_85256_MOESM2_ESM.docx]

Creation and validation of models to predict response to primary treatment in serous ovarian cancer

Jesus Gonzalez Bosquet,^1,2,^* Eric J. Devor,^3^ Andreea M. Newtson,^1^ Brian J. Smith ^2,4^, David P. Bender,^1,2^ Michael J. Goodheart,^1,2^ Megan E. McDonald,^1^ Terry A. Braun,^2,5^ Kristina W. Thiel,^3^ Kimberly K. Leslie^2,3^

^1^ Division of Gynecologic Oncology, Department of Obstetrics and Gynecology, University of Iowa Hospitals and Clinics, Iowa City, IA 52242, USA; [andreea-newtson@uiowa.edu](mailto:andreea-newtson@uiowa.edu) (A.M.N.); [megan-e-mcdonald@uiowa.edu](mailto:megan-e-mcdonald@uiowa.edu) (M.E.M.); [david-bender@uiowa.edu](mailto:david-bender@uiowa.edu) (D.P.B.); [michael-goodheart@uiowa.edu](mailto:michael-goodheart@uiowa.edu) (M.J.G.)

^2^ Holden Comprehensive Cancer Center, University of Iowa Hospitals and Clinics, Iowa City, IA 52242, USA

^3^ Department of Obstetrics and Gynecology, University of Iowa Hospitals and Clinics, Iowa City, IA 52242, USA; [kristina-thiel@uiowa.edu](mailto:kristina-thiel@uiowa.edu) (K.W.T.); [eric-devor@uiowa.edu](mailto:eric-devor@uiowa.edu) (E.J.D.); kimberly-leslie@uiowa.edu (K.K.L.)

^4^ Department of biostatistics, University of Iowa College of Public Health, Iowa City, IA 52242, USA; [brian-j-smith@uiowa.edu](mailto:brian-j-smith@uiowa.edu) (B.J.S.)

^5^ Coordinated Laboratory for Computational Genomics, University of Iowa Hospitals and Clinics, Iowa City, IA 52242, USA; [terry-braun@uiowa.edu](mailto:terry-braun@uiowa.edu) (T.A.B.)

* Correspondence: [jesus-gonzalezbosquet@uiowa.edu](mailto:jesus-gonzalezbosquet@uiowa.edu) (J.G.B.); Tel.: +1-(319)-356-2160

**SUPPLEMENTARY METHODS**

The objective of this section is to give some details about some of the methods and analyses performed in our study.

*Clinical variables:*

We introduced several types of clinical variables in the analysis of prediction: epidemiological, imaging, pathological, surgical and outcomes.

Epidemiological data: Age, BMI, Preoperative CA125, Charlson Comorbidity Score, Imaging by Ultrasound, Imaging by CT, Imaging by PET/CT scan, Imaging by MRI.

Imaging data from reports: Disease in Pelvis, disease in upper abdomen, disease in ovaries, disease in uterus, disease in bladder, disease in colon-rectum, disease in pelvic lymph nodes, disease in omentum, disease in colon-upper abdomen, disease in spleen, disease in porta-hepatis, disease in suprarenal lymph nodes, disease in mesenteric lymph nodes, disease in pleura, ascites in upper abdomen, disease in chest, other organs affected in upper abdomen.

Pathological data (all cases were reviewed to be serous type): FIGO Stage, histological grade.

Surgical data from reports: surgery to remove uterus, surgery to remove cervix, surgery to remove the adnexa (ovaries and tubes), surgery to remove omentum, surgery to remove pelvic lymph nodes, surgery to remove para-aortic lymph nodes, surgery to remove small bowel, surgery to remove colon/large bowel, surgery to remove diaphragm, surgery to remove spleen, surgery to remove other organs in upper abdomen, surgical complexity score.^1^

Outcome and treatment data: Response to therapy, neoadjuvant therapy, optimal surgery, optimal surgical outcome (including optimal surgery, mortality 90 days after surgery and start of chemotherapy before 2 months after surgery).^2^

*Biological Data:*

Quality control of RNA sequencing and DNA methylation:

Quality control (QC) of methylationEPIC arrays was performed with *shinyMethyl* and *Minfi* R packages.^3,4^ To assess outliers and possible technical biases be assessed: Bisulfite conversion, QC plot (**Supplementary Figure S1**), and Beta-values density (**Supplementary Figure S2**).


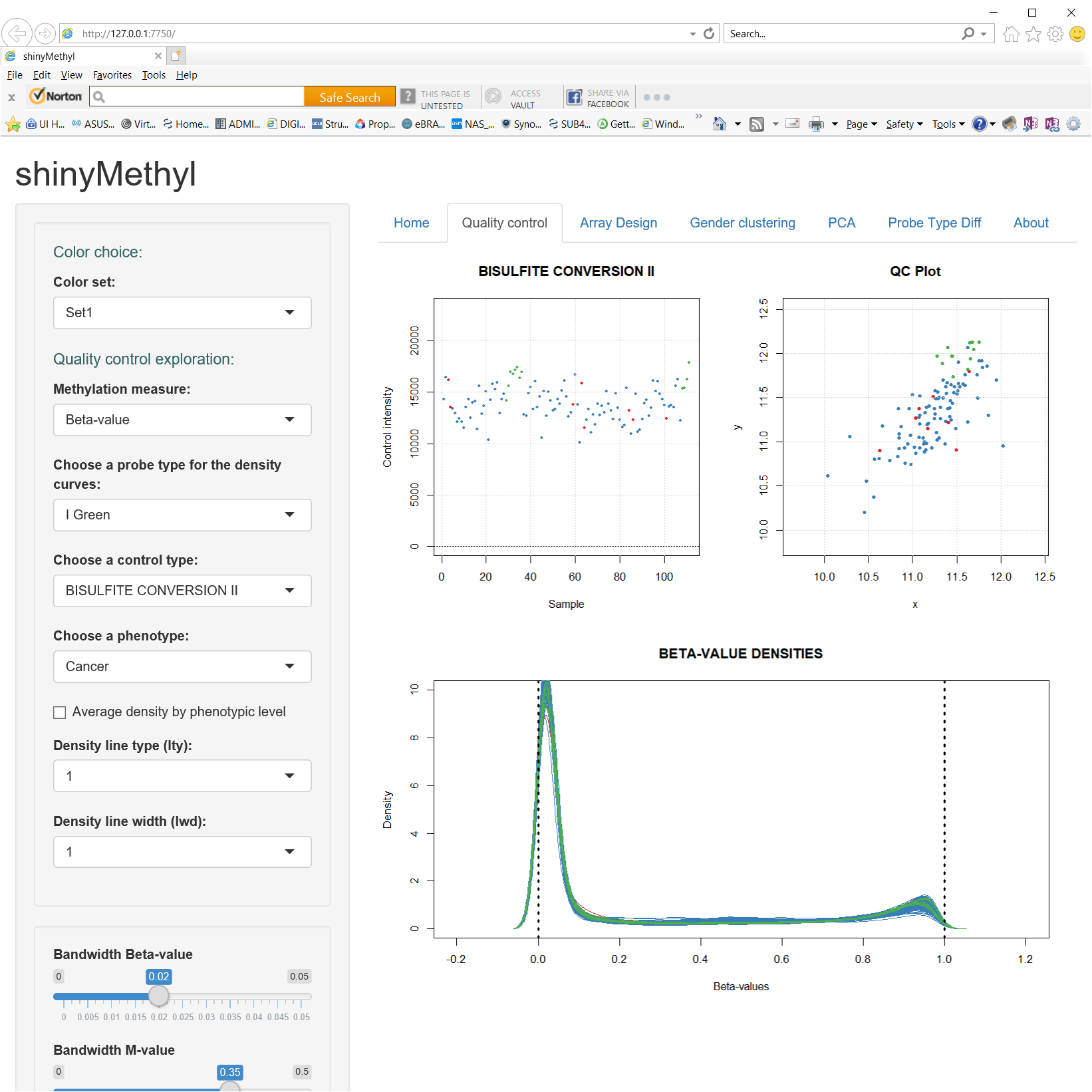


**Supplementary Figure S1**: QC of methylationEPIC arrays showed no obvious outliers for the different analyses performed. Cancer samples are in blue, tubal samples in green. Red represent cell cultures added to the study for control. Graphics were generated with R package *shinyMethyl*.^3^

**Supplementary Figure S2**: QC by methylation distribution for all samples. We expect to find a bimodal shape showing two peaks reflecting high number of low and high methylation. Graphics were generated with R package *shinyMethyl*.^3^

Also, we assessed QC by proportion of failed probes: 232 probes failed out of 866,091 for all experiments (probe failure of 2.68x10^-04^), which is below the 0.1 threshold.

For QC of our RNA-Seq experiments, we looked at the number of reads per sample and number of unmapped transcripts (see **Supplementary Table S1** for details). None of the samples had a number of mapped reads below the 10 million threshold.

**Supplementary Table S1:** Average of number of reads per sample for responders and non-responders. Also unmapped reads were computed for too many mismatches, for sequences too short (set up at length <200 bp, for 300 bp reads) and for other reads (as reported by STAR alignment software).

|  | Responders | Non-responders | p-value |
| --- | --- | --- | --- |
| **Number of input reads (mean)** | 28,364,017.06 | 27,124,213.97 | 0.539 |
| **Number of reads unmapped: too many mismatches (mean)** | 0 | 0 | -- |
| **Number of reads unmapped: too short (mean)(%)** | 6,423,659.88  (22%) | 5,729,251.36  (21%) | 0.274 |
| **Number of reads unmapped: other (mean)(%)** | 63,307.62  (0.2%) | 51,746.65  (0.2%) | 0.243 |

Pre-processing of biological data

A flow diagram of how different genomic data was extracted from fastq and BAM files is represented in **Supplementary Figure S3.**


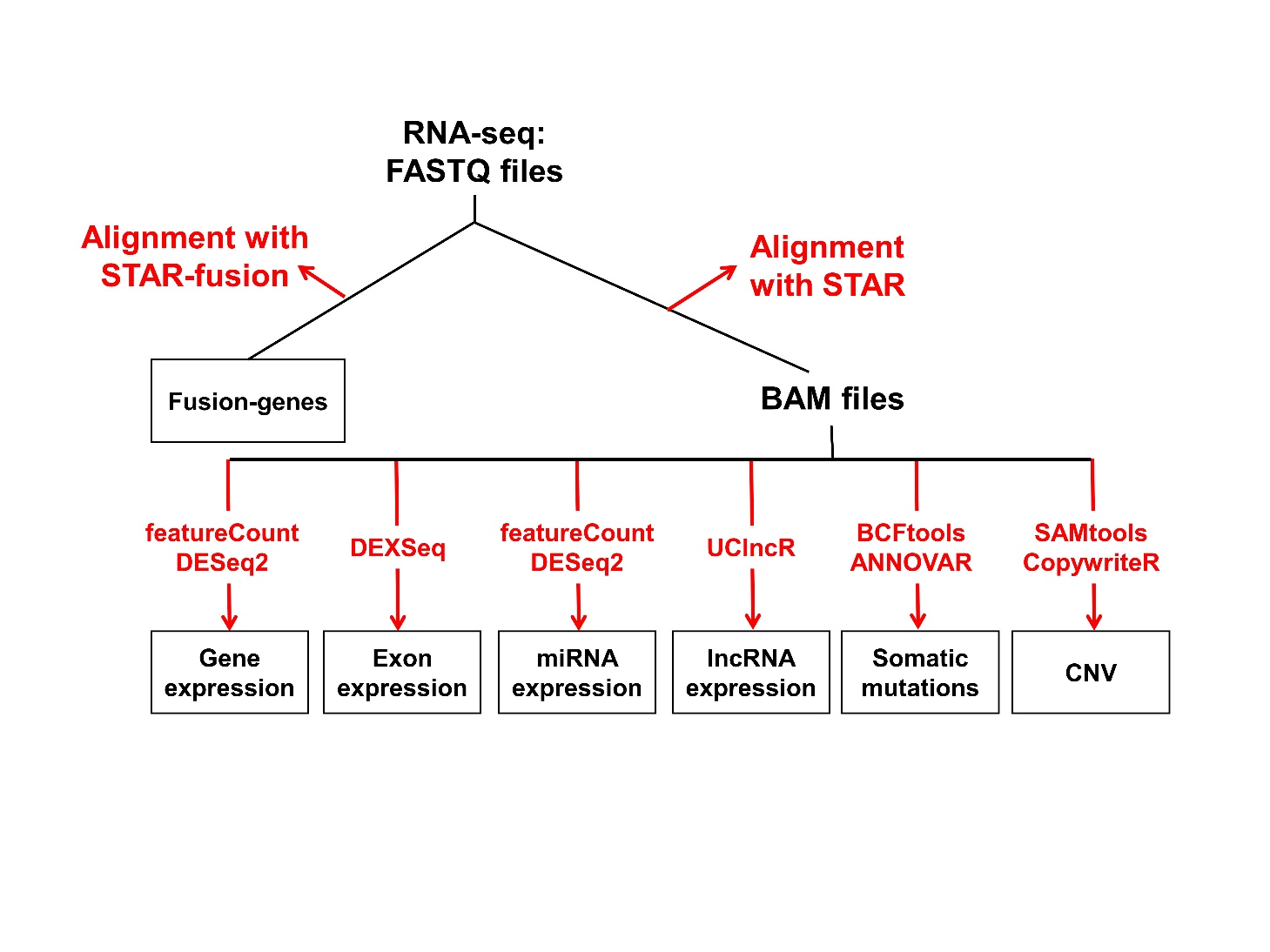


**Supplementary Figure S3: Pipeline of genomic analytics starting with RNA sequencing.**

From *fastq* files originated from RNA-seq, we created fusion genes and BAM files. The rest of genomic elements were produced from these BAM files and different software analytics. In red, different software utilities used to generate genomic elements for this project.

*Statistical analysis*

*Validation - TCGA Data:*

The following clinical variables were available in TCGA dataset for validation of UI models of treatment response prediction: Response to therapy, Age, neoadjuvant therapy, and optimal surgery.

**Supplementary Table S2: HGSC TCGA patient characteristics and association with treatment response**

|  | | **Responders** | **Non-responders** | **p-value** |
| --- | --- | --- | --- | --- |
|  |  | **N=189** | **N=149** |  |
| **Age** |  | **58** | **59** | **0.337** |
| **Stage** | **3** | **162** | **121** | **0.397** |
|  | **4** | **27** | **26** |  |
| **Grade** | **2** | **22** | **19** | **0.739** |
|  | **3** | **167** | **130** |  |
| **Residual disease after surgery** | **Optimal (<1 cm)** | **136** | **85** | **<0.001** |
|  | **Suboptimal (>1 cm)** | **31** | **58** |  |
| **Neoadjuvant Chemotherapy** | **Yes** | **0** | **0** | **NA** |
|  | **No** | **189** | **149** |  |

NA: non-applicable

*Validation - Analysis:*

This analysis, was performed with R packages *caret*, *gmlnet*, and *pROC*, and provided a predicted probability for treatment response for each patient that ranges from 0 (responder) to 1 (non-responder).^5-7^ We used these analytics to determine thresholds, or cut-offs, for the UI model applied to TCGA data.^8^ Specifically, we determined a *threshold* value for model probabilities whereby values above the threshold will be classified as non-responders and below as responders.

The threshold parameter was treated as a tuning parameter for which values sought to produce a final classification model. A grid of tuning parameters was considered (e.g. all combinations of thresholds from 0 to 1 in steps of 0.01). At each grid point, sensitivity and specificity was estimated. Tuning parameters that yield sensitivity >90% were ranked from highest to lowest specificity. Note that by placing a restriction on sensitivity and not specificity, we guaranteed to identify tuning parameters in the desired range. For instance, a threshold probability value at the extreme of 0 will classify all subjects as non-responders and thus have a sensitivity of 100%. Among the ranked results, the top-ranked set of tuning parameters were used to fit a final model to the entire set of patients and define the classification rule for identifying non-responders. An example of an hypothetical UI-built model validated in TCGA data is represented in **Supplementary Figure S4**.


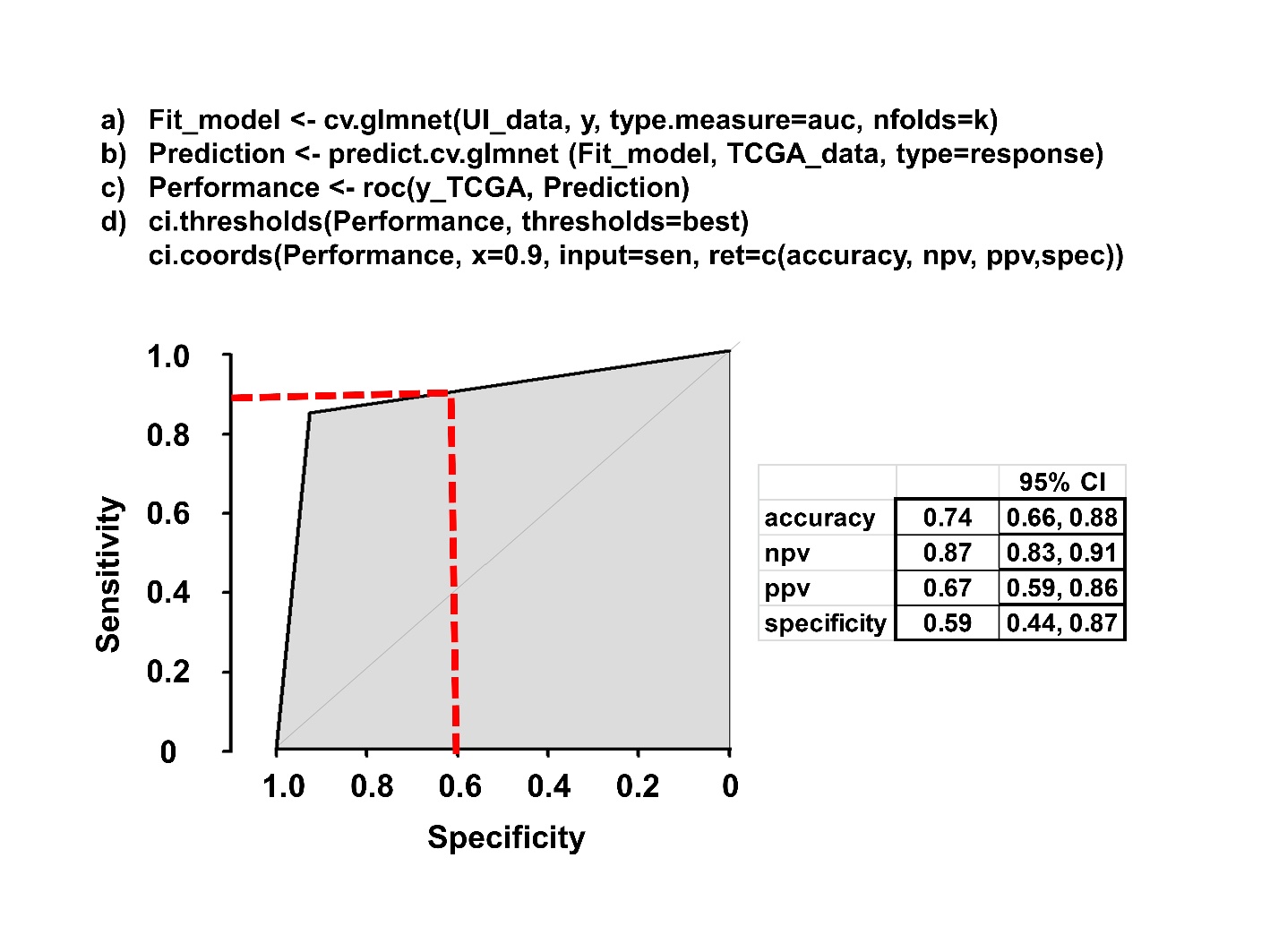
Supplementary Figure S4: Validation of UI prediction models in independent datasets. Summary of model creation and validation: a) First we created the model (*Fit_model*) using lasso (*cv.glmnet*) and UI data (*UI_Data*); *y* are the labels: responders or non-responders; the *measure* is AUC; the model is built with k-fold cross-validation (*nfolds*). b) Then we applied the created model (*Fit_model*), with the data of the independent dataset (in this example *TCGA_data*); and created a predicted probability for response to treatment for each patient (*Prediction*). c) Then we determined the performance of this prediction (*Performance*) comparing it with the real labels in the independent database (y_TCGA). d) To select the thresholds for the predictor (*ci.threshold*) we used the best threshold based on pre-established algorithms, or use a cut-off of interest, in this case sensitivity (*sen*) of 90%, that gave us parameters to measure the performance of the model (*ci.coords*) with specificity (*spec*), negative predictive value (*npv*), positive predictive value (*ppv*), and *accuracy*.

The graphic represents a hypothetical validation of the model with an AUC of 89% (area in grey) using a threshold of 90% sensitivity (horizontal red dotted line), resulting in 59% specificity (vertical red dotted line).

*Validation - Adjustment:*

In previous studies we observed that genetic background, or admixture, was different between UI and TCGA populations and that may affect the performance of the validation.^9^ In preliminary validation analyses we noticed that models with clinical data may be affected by this genetic background (**Supplementary Fig. S5**).

**
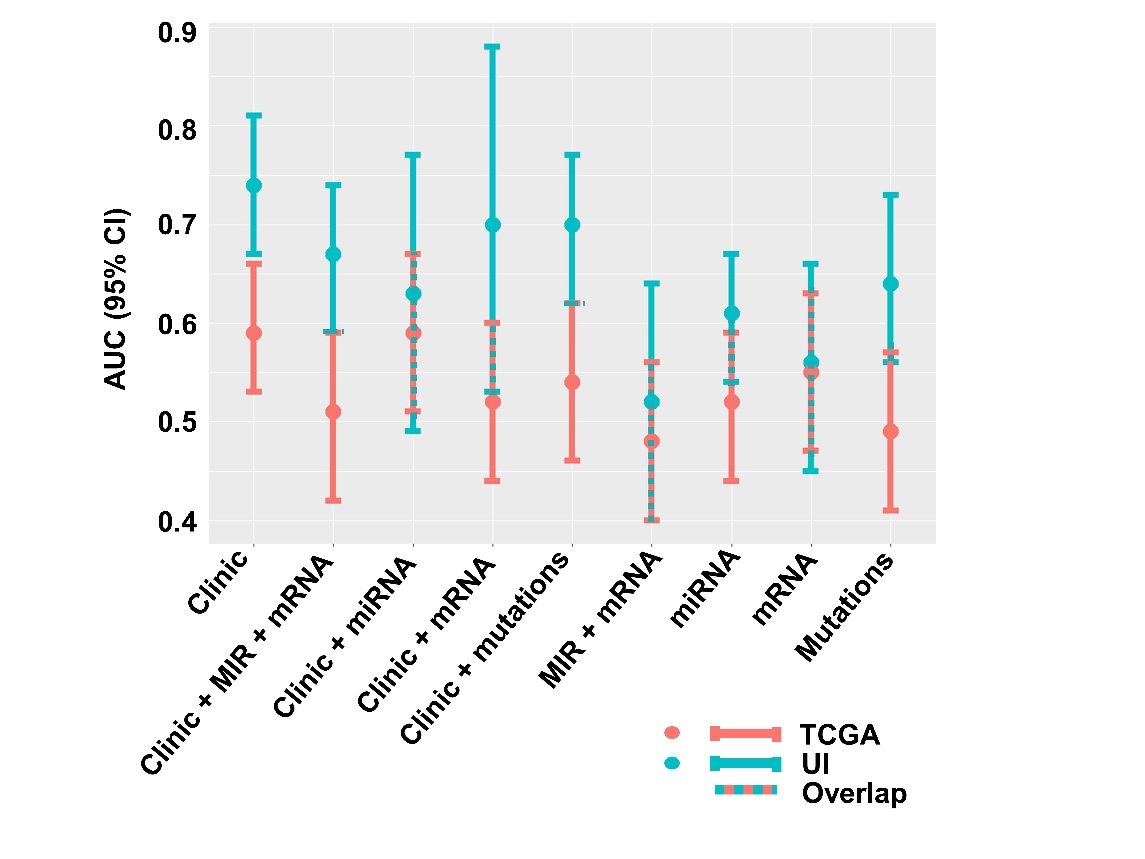
**

**Supplementary Figure S5: Validation of UI prediction models in TCGA.** UI prediction models of treatment response were validated in TCGA and compared in terms of area under the curve (AUC) with their 95% confidence intervals (CI). There was some degree of 95% CIs overlap in all models, except for the clinical model.

We designed two strategies to adjust or account for possible genetic background differences between both population of patients to adjust. First, we extracted genotypes for both populations, UI and TCGA. Briefly, BAM files were obtained from RNA-seq alignments to the hg38 human genome version. Then, they were converted to VCF files (variant call format file) for genotype extraction. *PLINK* software was then used to filter the VCF files by minor allele frequency at q > 0.05 and by linkage disequilibrium (LD) < 0.1 r^2^ for pairs of markers inside 200 kb. We imputed missing genotypes with *Beagle 4.1*.^10^ The filtering process was performed to identify independent markers within the sequenced samples and resulted in 233,160 markers for both UI and TCGA. Then we applied two different strategies:

1. Principal component analysis (PCA) was performed with all genotypes and all samples (UI=88 and TCGA=218) to identify principal components (PC) that would differentiate one population from the other (UI from TCGA). Then we used the first 3 PC as predictors in the model to adjust for the 2 different populations (**Supplementary Fig. S6A**).
2. Lasso regression analysis was performed with all genotypes and all samples to identify the markers that predicted being part of TCGA cohort. Resulted in 4 markers that classify TCGA cohort with an AUC of 100% (**Supplementary Fig. S6B**). Then we used these 4 markers as predictors in the model to adjust for the 2 different populations.

While there is no overlap (differences) between the AUC 95% CI the UI model and TCGA validation model without adjustment (first row in left panel of **Supplementary Fig. S6C**), or when we adjust with the first 3 PC (second row in left panel of **Supplementary Fig. S6C**); when we adjusted with the resulting 4 genotypes that discriminate TCGA versus UI population, there was a wide AUC 95% CI overlap (or no difference).

**
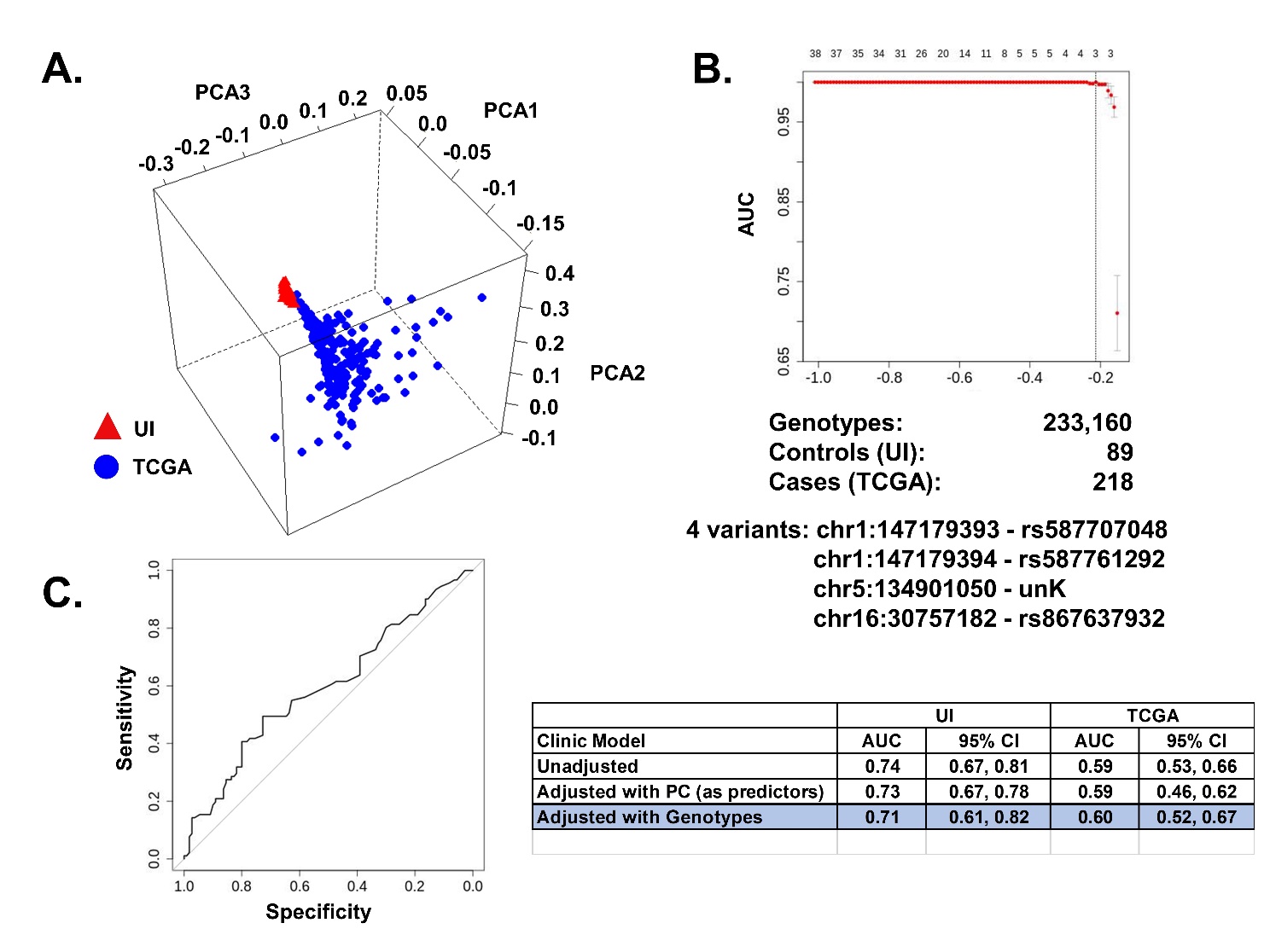
**

**Supplementary Figure S6: TCGA validation of clinical model.**

**A.** *Principal component analysis (PCA) of TCGA and UI genotypes*: BAM files were obtained from RNA-seq alignments and converted to VCF files for genotype extraction. PCA was performed with all genotypes. In the 3D plot the first 3 PC are represented: UIHC samples are grouped very close (red triangles); TCGA samples are more dispersed (blue circles). Graphics were generated with R package *lattice.*^11^ **B.** *Lasso regression* analysis to identify the makers that predicted being part of TCGA cohort: resulted in *4 markers* that classify TCGA cohort with an AUC of 100%. Three of these markers are known SNPs, the fourth is a variant of unknown significance (unK). **C.** *Validation of Clinical Model of UI in TCGA data*: on the right panel are different validations based on their adjustment: First, with no adjustment (95% CI do not overlap); second, with PC adjustment as predictors (no overlap); third, with adjustment of 4 more representative genotypes for TCGA, with overlap of 95% CI (highlighted in blue). On the left panel a ROC curve of this last validation with genotype adjustment.

**ADITIONAL REFERENCES**

1. Aletti GD, Dowdy SC, Podratz KC, Cliby WA. Relationship among surgical complexity, short-term morbidity, and overall survival in primary surgery for advanced ovarian cancer. Am J Obstet Gynecol 2007;197:676 e1-7.

2. Newtson AM, Devor EJ, Gonzalez Bosquet J. Prediction of Epithelial Ovarian Cancer Outcomes With Integration of Genomic Data. Clin Obstet Gynecol 2020;63:92-108.

3. Fortin JP, Fertig E, Hansen K. shinyMethyl: interactive quality control of Illumina 450k DNA methylation arrays in R. F1000Res 2014;3:175.

4. Aryee MJ, Jaffe AE, Corrada-Bravo H, et al. Minfi: a flexible and comprehensive Bioconductor package for the analysis of Infinium DNA methylation microarrays. Bioinformatics 2014;30:1363-9.

5. Robin X, Turck N, Hainard A, et al. pROC: an open-source package for R and S+ to analyze and compare ROC curves. BMC Bioinformatics 2011;12:77.

6. Kuhn M. Building Predictive Models in R Using the caret Package. J Stat Softw 2008;28:1-26.

7. Friedman J, Hastie T, Tibshirani R. Regularization Paths for Generalized Linear Models via Coordinate Descent. J Stat Softw 2010;33:1-22.

8. Sing T, Sander O, Beerenwinkel N, Lengauer T. ROCR: visualizing classifier performance in R. Bioinformatics 2005;21:3940-1.

9. Miller MD, Devor EJ, Salinas EA, et al. Population Substructure Has Implications in Validating Next-Generation Cancer Genomics Studies with TCGA. Int J Mol Sci 2019;20.

10. Browning BL, Browning SR. Genotype Imputation with Millions of Reference Samples. Am J Hum Genet 2016;98:116-26.

11. Sarkar D. Lattice : multivariate data visualization with R. New York: Springer; 2008.
