# Supplementary material for: Creation and validation of models to predict response to primary treatment in serous ovarian cancer
Source: Sci Rep. 2021 Mar 16;11:5957. doi: 10.1038/s41598-021-85256-9 (PMC7971042; doi:10.1038/s41598-021-85256-9)

**Supplementary Figure S7: Performances of all prediction model of response (N=107).**

On the left is the number of types of data. For 1 type of data, ‘Clinical data’ is highlighted for reference. Different performances are displayed in ascending order. The x axis is AUC as a percentage (0-100%). There are several combinations of 2 and 3 types of data with performances over 95% AUC. SNV: single nucleotide variation; CNV: gene copy number; DEXSeq: exon expression; lncRNA: long non-coding RNA; MIR: micro RNA, mRNA: gene expression. Graphics were generated with R package *ggplot*.^69^


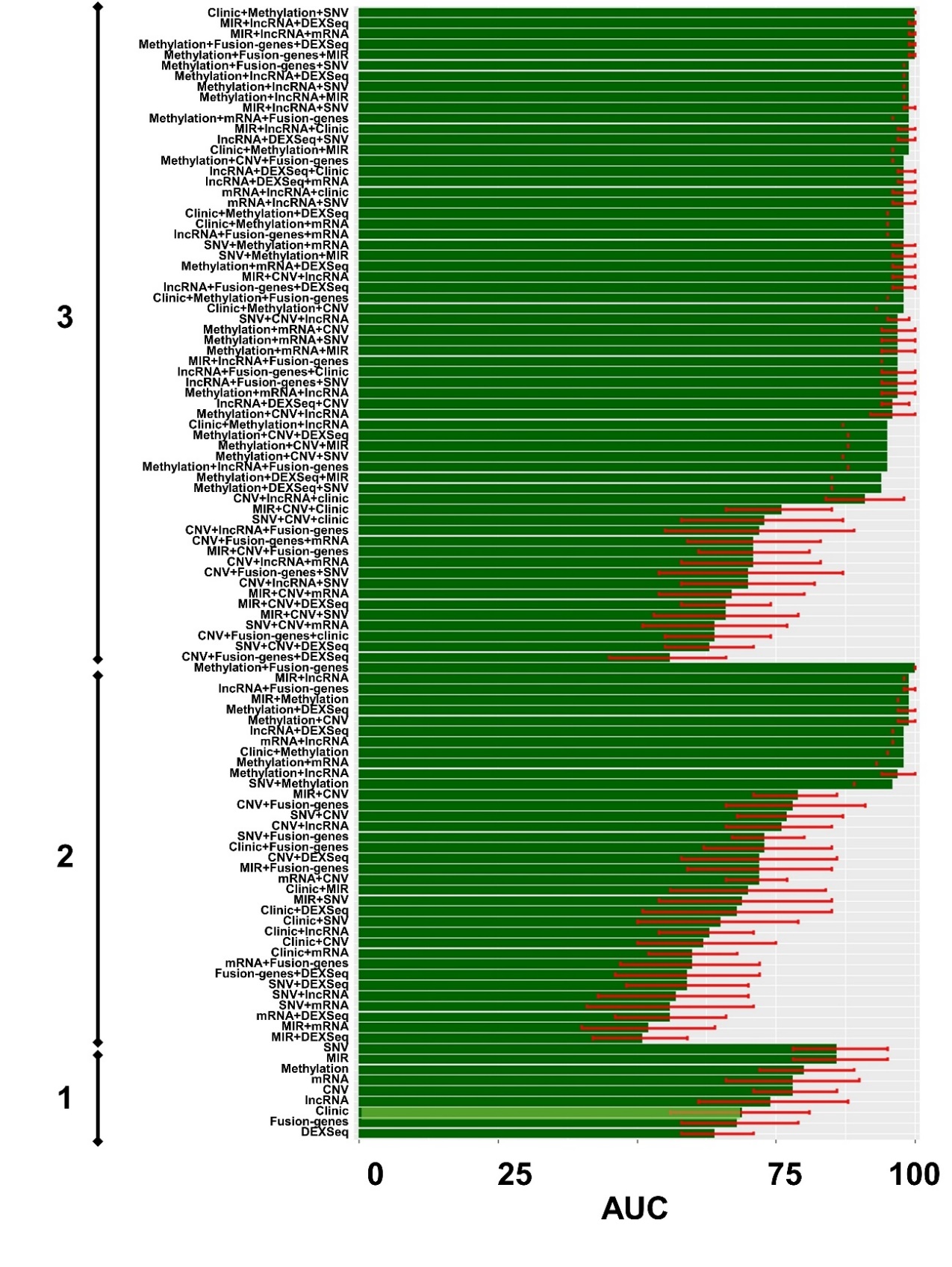

Supplement: Supplementary file 3 — Supplementary Information 3. [file 41598_2021_85256_MOESM3_ESM.docx]
